# Heat‐treated egg allergens show lower basophil activation: A path toward safer oral immunotherapy

**DOI:** 10.1111/pai.70275

**Published:** 2026-01-05

**Authors:** Marta Paolucci, Maria Breiding, Macsmeila Dietrich, Jana G. Meyer, Agathe Duda, Alice Köhli, Claudia C. V. Lang, Sayeh Agah, Sabina Wuenschmann, Klaus Eyer, Thomas M. Kündig, Johannes Trück, Pål Johansen

**Affiliations:** ^1^ Department of Dermatology University of Zurich Zurich Switzerland; ^2^ Division of Allergy, University Children's Hospital and Children's Research Center University of Zurich Zurich Switzerland; ^3^ Laboratory for Functional Immune Repertoire Analysis, Institute of Pharmaceutical Sciences, D‐CHAB ETH Zurich Zurich Switzerland; ^4^ Department of Dermatology University Hospital Zurich Zurich Switzerland; ^5^ Department of Paediatrics Hospital Baden Baden Switzerland; ^6^ InBio Charlottesville Virginia USA; ^7^ Department of Biomedicine Aarhus University Aarhus Denmark

**Keywords:** basophil degranulation test, circular dichroism spectroscopy, egg hypersensitivity, egg proteins, food hypersensitivity, immunoglobulin E, ovalbumin, ovomucoid, pediatrics, protein denaturation

## Abstract

**Background:**

Oral immunotherapy (OIT) is a promising approach for treating IgE‐mediated food allergy, but safety concerns limit its use. Heat‐denaturation of food allergens may reduce allergic reactions by lowering IgE binding. Here, we examined how heat‐induced structural changes in egg allergens affected basophil activation in egg‐allergic patients.

**Methods:**

Gal d 1 and Gal d 2 were subjected to heat treatment and analyzed for structural changes using SDS‐PAGE, ELISA, NanoDSF, and circular dichroism. Peripheral blood samples were obtained from a cohort of 42 patients with egg allergy. Patients' sensitization status was determined, and basophils were isolated and incubated with native or heat‐denatured egg allergen preparations. Basophil activation was assessed by measuring leukotriene release as a marker of degranulation.

**Results:**

Heat‐denaturation induced time‐ and temperature‐dependent structural changes in both Gal d 1 and Gal d 2, resulting in reduced IgE binding capacity. In functional assays, heat‐denatured allergens elicited weaker basophil degranulation responses compared to native allergens, but the effect varied depending on individual IgE sensitization profiles. Among patients who reacted to heat‐denatured allergens, egg‐white IgE levels tended to be higher, although requiring higher doses to trigger leukotriene release.

**Conclusion:**

Heat‐denaturation of egg allergens reduces IgE‐binding and basophil activation, although residual reactivity persists in patients with higher sensitization profiles. Importantly, higher allergen doses were needed to trigger basophil degranulation compared to native allergens, indicating a reduction in allergenic potency. These findings highlight the potential of heat‐denatured egg allergens as safer starting materials for OIT, particularly within personalized, stepwise desensitization protocols, warranting further clinical investigation.

**Key message**

This study provides a comprehensive analysis of how heat processing alters the structural and immunological properties of major egg allergens, Gal d 1 and Gal d 2, using both purified proteins and complex food matrices. Our findings contribute to the understanding of how thermal processing reduces allergenicity, supporting the development of safer food products and informing dietary management strategies for patients with egg allergy. The work is highly relevant to clinicians, allergists, and food scientists as it bridges molecular allergology and practical implications for allergy diagnosis, food manufacturing, and immunotherapy approaches.

## 1 INTRODUCTION

Food allergies are IgE‐mediated reactions driven by Th2‐dominated immune responses, in which allergen binding to IgE on the surface of basophils and mast cells triggers their degranulation and the subsequent release of inflammatory mediators, leading to allergic symptoms.^1^ Food allergies are also steadily rising, affecting up to 9% of children and 4% of adults worldwide.^2^ Currently, dietary avoidance remains the primary management strategy.^3^ Targeted treatment options are highly limited, with Palforzia the only approved allergen‐specific immunotherapy for peanut allergy, and anti‐IgE omalizumab offering an allergen non‐specific approach.^4^ However, due to the widespread presence of allergenic foods, frequent food cross‐contamination, as well as allergen cross‐reactivity, strict avoidance is challenging, and accidental ingestion is common.^5^

Various immunotherapy approaches are being explored for the treatment of food allergies, including oral, sublingual, and epicutaneous routes, with oral immunotherapy (OIT) emerging as a particularly promising option. The goal of OIT is to raise the threshold for allergic reactions following accidental exposure, that is, desensitization.^6^, ^7^ OIT induces immune modulation by shifting the response from Th2 dominance toward regulatory T cells and Th1 cells, promoting the production of immunosuppressive cytokines and allergen‐specific IgG4, thereby reducing IgE‐mediated allergic reactions.^8^, ^9^, ^10^

Egg allergy is one of the most prevalent food allergies, particularly in children,^7^, ^11^, ^12^ and although OIT with egg allergens has come further than OIT with other allergens, the effect of egg OIT in children is still inconclusive.^2^, ^13^, ^14^, ^15^, ^16^, ^17^, ^18^, ^19^ While OIT often leads to successful desensitization, remission or sustained unresponsiveness remains less common.^7^ Moreover, OIT is not without risks, as the administration of allergenic proteins can trigger adverse reactions at any stage of treatment.^7^, ^20^ These reactions can range from mild and local oropharyngeal itching or temporary abdominal discomfort to anaphylaxis, all of which may lead to OIT discontinuation.^2^, ^21^, ^22^ Notably, OIT has been linked to a higher frequency of anaphylactic reactions and an increased need for epinephrine compared to strict allergen avoidance.^2^

Egg allergy is primarily mediated by IgE responses to proteins in egg white, including Gal d 1 (ovomucoid), Gal d 2 (ovalbumin), Gal d 3 (conalbumin), and Gal d 4 (lysozyme), which together constitute approximately 80% of total egg white protein content.^23^, ^24^ The allergenic potential of these proteins is significantly influenced by their resistance to heat and enzymatic digestion.^7^ Heat treatment or partial hydrolysis can alter the tertiary structure of proteins, disrupting conformational epitopes without affecting linear epitopes, potentially reducing IgE binding.^25^, ^26^, ^27^ Gal d 1 is known to be a highly potent allergen with a stronger anaphylactic potential than other egg allergens, also due to its resistance to heating and enzymatic digestion.^28^ In contrast, Gal d 2, 3, and 4 are heat‐labile proteins that are readily denatured.^24^, ^29^ Moreover, sensitization to Gal d 1 is a strong predictor of clinical reactivity to both raw and heated egg, posing a higher risk for persistent egg allergy.^24^, ^30^, ^31^

To enhance the safety and efficacy of OIT, heat‐denaturation has been explored as a potential approach.^32^, ^33^, ^34^, ^35^, ^36^ Tolerance to baked egg is often achieved faster than to raw egg,^7^, ^37^ and approximately 70% of children with egg allergy can tolerate egg in its baked form; hence, introducing small amounts of baked eggs at an early stage may lower the likelihood of allergic sensitization.^2^, ^38^ Moreover, OIT using baked egg has been shown to cause desensitization even in children who initially did not tolerate baked egg, while also increasing their tolerance to raw egg.^39^, ^40^, ^41^

This study aimed to evaluate the effects of heat‐denaturation on the major egg allergens, Gal d 1 and Gal d 2, to assess their potential as safer options for allergen immunotherapy (AIT) in individuals with egg allergy. Specifically, we examined the structural changes and IgE reactivity of native and heat‐denatured egg allergens. We also evaluated the safety potential of heat‐denatured allergens by using a leukotriene release assay in a cohort of egg‐allergic patients. Given the availability and production of allergen extracts, along with the simplicity of heat‐denaturation, the results support the potential use of heat‐denatured egg allergens as a safer material in OIT.

## 2 MATERIALS AND METHODS

### 2.1 Heat denaturation of Gal d 1 and Gal d 2 proteins

Purified natural Gal d 1 (ovomucoid, LoTox natural Gal d 1, LTN‐GD1‐1, InBio, Charlottesville, VA) and natural Gal d 2 (ovalbumin, LoTox natural Gal d 2, LTN‐GD2‐1, InBio) were diluted to 1 mg/mL in 1.5 mL Eppendorf tubes and heated at 20°C–100°C for 10 min to 6 h using a water bath or a thermo‐shaker. Samples were then chilled in an ice bath for 1 min to halt denaturation. Both heat‐denatured and native controls were stored at 4°C until further use.

### 2.2 Preparation of whole egg powder (WEP) solution

Pasteurized whole egg powder (WEP; Rembrandt Foods, Rembrandt, IA) was reconstituted in sterile 0.9% saline at 10 mg/mL in a 50 mL Falcon tube. Protein extraction was performed by gentle rotation at room temperature under aseptic conditions. The solution was diluted to 1 mg/mL in sterile 0.9% saline without vortexing to prevent foaming. After centrifugation at 3220 rcf for 10 min, supernatants were collected and aseptically aliquoted for storage at 4°C (short‐term) or −20°C (long‐term).

### 2.3 Heat denaturation of WEP

Aliquots of 1 mg/mL WEP were incubated at 65 °C, 75 °C, 85°C, or 95°C for 10 or 20 min; additional samples were incubated at 95 °C for 1 h. Heating was performed in a water bath or thermo‐block with mild agitation (500 rpm), followed by rapid cooling on ice. Samples were centrifuged at 3220 rcf for 10 min, and supernatants stored at 4 °C or −20 °C. Notably, heating at 65°C–75 °C for 10–20 min aligns with common low‐temperature pasteurization protocols.

### 2.4 Sodium Dodecyl Sulfate PolyAcrylamide Gel Electrophoresis (SDS‐PAGE)

Ten micrograms of Gal d 1 or Gal d 2 were mixed with sodium dodecyl sulphate (SDS) loading buffer (non‐reducing) and heated at 95 C for 5 min. Samples were resolved on 4%–20% Mini‐PROTEAN TGX precast gels (Bio‐Rad, Hercules, CA) using SDS running buffer. Gels were stained with InstantBlue® Coomassie (Abcam, Waltham, MA), destained in deionized water, and imaged using a Gel Doc EZ Imager and Image Lab software (Bio‐Rad).

### 2.5 Enzyme‐linked immunoassay (ELISA) for egg allergen quantification

To assess the impact of heat treatment on purified Gal d 1, Gal d 2, and WEP preparations, allergen concentrations were measured using commercial ELISA kits (EPC‐GD1 and EPC‐GD2, InBio), following the manufacturer's instructions.

### 2.6 Enzyme‐linked immunoassay (ELISA) for egg allergen IgE binding

To assess IgE binding to Gal d 1, a chimeric ELISA was performed.^42^ Microtiter plates were coated with native or heat‐denatured Gal d 1 (95 °C for 2 or 6 h; InBio) at 0.1 μg/well overnight at 4 °C. Wells were incubated for 1 h with plasma from three egg‐allergic individuals (PlasmaLab, Everett, WA) at 1:2 and 1:10 dilutions in PBS with 0.05% Tween‐20 and 1% bovine serum albumin. Bound human IgE was detected with peroxidase‐labeled goat anti‐human IgE (SeraCare, Milford, MA) and quantified using a chimeric mouse‐human IgE standard curve.

IgE binding to Gal d 2 was measured via sandwich ELISA. MaxiSorp 96‐well plates were coated with mouse anti‐ovalbumin IgE (1:2000, MCA2259, Bio‐Rad) in carbonate buffer and incubated overnight at 4 C. After blocking with 5% skimmed milk in PBS and Tween20 (PBSTM), 50 μL of Gal d 2 (20 μg/mL) in PBSTM was added and incubated for 1 h. HRP‐conjugated rabbit anti‐ovalbumin (1:2000, Invitrogen) was then added for 1 h. After final washing, 50 μL TMB substrate was added and stopped with 25 μL 2 N H₂SO_4_. Absorbance was read at 450 nm using a Spark 10 M reader (Tecan, Maennedorf, Switzerland).

### 2.7 Circular dichroism (CD) spectroscopy

Secondary structure analysis was performed using a Chirascan VX spectrometer (Applied Photophysics, Leatherhead, UK) with allergen samples at 0.1 mg/mL in a 0.10 cm pathlength cuvette. Replicate spectra (*n* = 5) were recorded from 190 to 280 nm at temperatures from 20 °C to 90 °C in 10 °C increments. Data were corrected for baseline, smoothed using a Savitzky–Golay filter, and reported as molar ellipticity [θ].^43^

### 2.8 Nano differential scanning fluorimetry

Protein stability was assessed by NanoDSF (Tycho NT.6, NanoTemper Techn, Munich, Germany). Gal d 2 at 0.1 mg/mL was scanned from 35 C to 95 C, and thermal unfolding was tracked via the 330/350 nm fluorescence ratio to determine the inflection temperature (Tm).

### 2.9 Patients cohort

A total of 42 patients diagnosed with egg allergy were recruited from three centres in Switzerland: Children's Hospital Zurich (KISPI), *Kantonsspital Baden* (KSB), and University Hospital Zurich (USZ). Both adult and pediatric patients of any gender or ethnicity were included. The inclusion criteria in children were oral or systemic reactivity to egg‐containing foods as confirmed by the allergists based on a consistent clinical history of reactions to cooked, baked, scrambled, or raw egg as reported by patients or parents. In three children and in two adults, the diagnosis was additionally confirmed by a physician‐supervised oral food challenge (OFC). The inclusion criteria in adults were a documented history of reaction to egg‐containing foods and a positive skin prick test. A further inclusion criterion was a signed written informed consent for subsequent use of blood samples by patients or by parents/legal guardians in case of children. Exclusion criteria included refusal or withdrawal of consent, medical contraindications to blood withdrawal, for example, anemia, recent systemic immunosuppressive treatment as well as antihistamines and mast cell stabilizers within the last 4 days before blood sampling, use of omalizumab or other biologics for treatment of allergy or asthma, and pregnancy or lactation. All blood samples were obtained during routine clinical visits to minimize burden.

### 2.10 ImmunoCAP

Total IgE, egg white‐specific IgE (f1), and Gal d 1‐specific IgE (f233) were quantified using the ImmunoCAP system on a Phadia 250 analyzer (Thermo Fisher Scientific, Waltham, MA). Samples were processed per the manufacturer's protocol, and IgE values were expressed in kUA/L and classified by RAST: <0.35 (negative), 0.35–0.70 (low), 0.7–3.50 (moderate), 3.50–17.50 (high), and ≥17.50 (very high).

### 2.11 Leukotrienes release assay

Leukotriene release was assessed to measure the primary functional endpoint because IgE‐dependent basophil activation triggers both surface activation markers and downstream mediator secretion. Whole blood was treated with dextran within 24 h of blood collection to sediment erythrocytes for 90 min. The leukocyte‐rich plasma was collected, and leukocytes were isolated by brief centrifugation. Cells were stimulated at 37 C for 40 min with native or heat‐denatured WEP. Because blood volume was limited in some of the younger children, sometimes <2 mL, different WEP concentrations and replicate numbers were used depending on the available sample volume. After centrifugation, supernatants were collected, and cysteinyl leukotrienes (LTC_4_, LTD_4_, LTE_4_) were measured using the CAST ELISA kit (Bühlmann Laboratories, Schoenenbuch, Switzerland). A positive response was defined as leukotriene release ≥2‐fold above baseline consistent with commonly used criteria in mediator‐release and basophil activation assays. To minimize false positives, each sample included an unstimulated control and replicate wells (*n* = 2–4) with predefined precision targets. Initially, we also included stimulation with irrelevant allergens (Fel d 1 and grass pollen extracts) to demonstrate allergen specificity.

### 2.12 Statistical analysis

Data were analyzed using GraphPad Prism v9 or R Studio v4.3.3. IgE data are shown as medians with 95% confidence intervals, other results as mean ± SEM. Comparisons between two groups used the Mann–Whitney *U* test. Pearson's correlation was used to assess relationships between IgE levels, age, and skin test size. Statistical significance was set at *p* < .05.

### 2.13 Ethics

The study complied with the Declaration of Helsinki and was approved by the Ethics Committee of the Canton of Zurich (BASEC 2023‐01405). Written informed consent was obtained from all participants or their legal guardians.

## 3 RESULTS

### 3.1 Heat treatment induces structural and functional changes of Gal d 1 and Gal d 2

**FIGURE 1:**
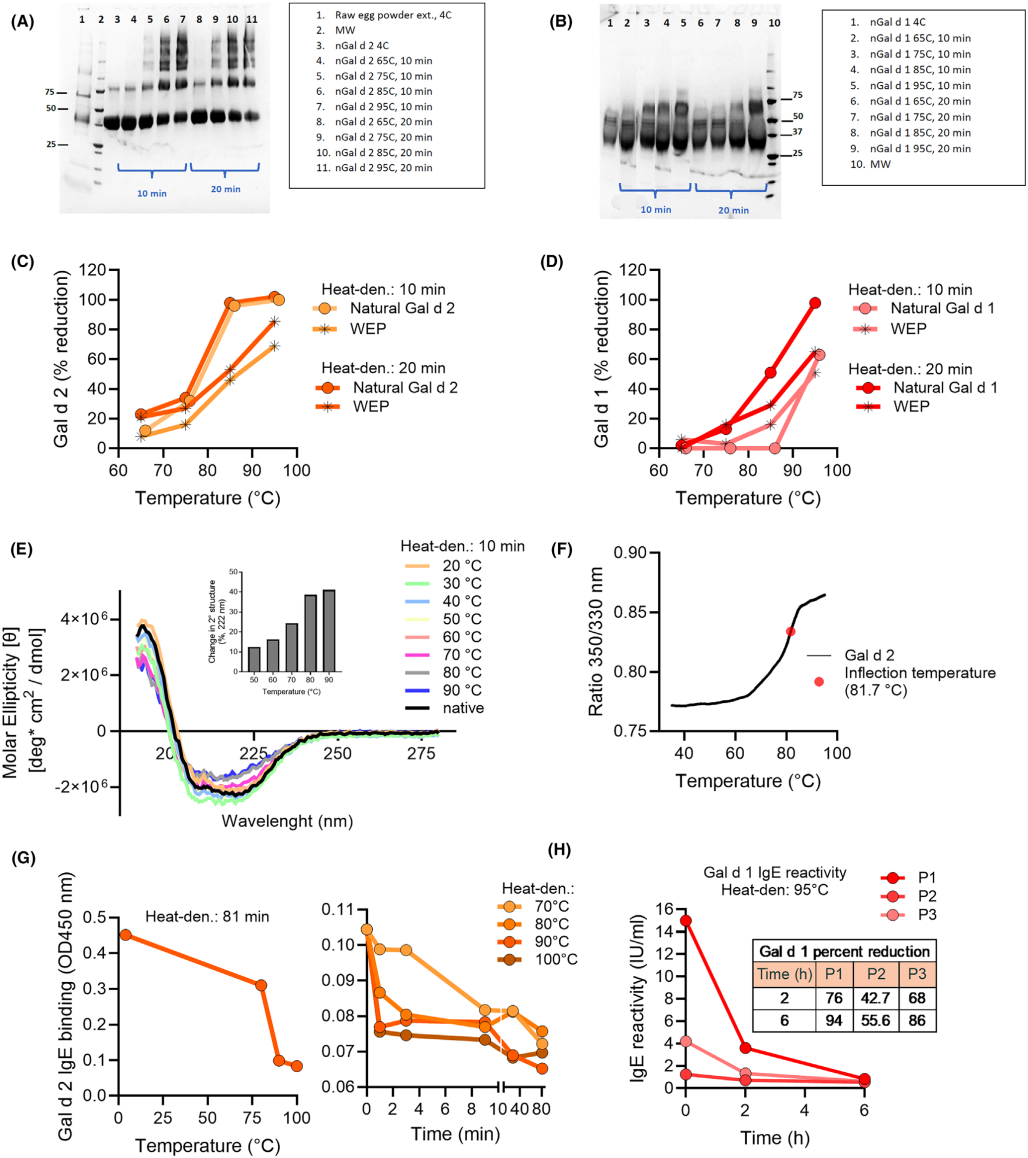
Impact of heat treatment on Gal d 1 and Gal d 2: structural and functional changes. SDS‐PAGE analysis of natural Gal d 2 (A) and Gal d 1 (B) following heat treatment at 65°C, 75°C, 85°C, and 95°C for 10 and 20 min. Untreated samples stored at 4°C served as controls. A raw egg powder extract was included in panel A for comparison. MW = molecular weight markers. ELISA analysis of natural Gal d 2 (C) and Gal d 1 (D), as well as Gal d 2 and Gal d 1 in whole egg powder (WEP) following heat treatment. Results are expressed as the percentage reduction in ELISA signal compared to the untreated control (4°C: 784 μg/mL Gal d 2 and 570 μg/mL Gal d 1), following heat exposure at 65°C, 75°C, 85°C, and 95°C for 10 and 20 min. (E) Circular dichroism spectrum (CD) of Gal d 2 obtained at temperatures ranging from 20°C to 90°C. Inset: Calculated percentage change in total secondary structure of Gal d 2 at 222 nm across temperatures from 50°C to 90°C, relative to Gal d 2 at 30°C. (F) Nano differential scanning fluorimetry of Gal d 2, indicating an inflection temperature of 81.7°C. (G) IgE binding activity curves for native and heat‐denatured Gal d 2, assessed by ELISA after exposure to various temperatures and incubation times. (H) Chimeric IgE ELISA analysis of denatured Gal d 1 and control using sera from egg‐allergic individuals (P1, P2, and P3). IgE reactivity is shown in IU/mL. In the table, the percentage reduction in chimeric IgE ELISA signal is indicated relative to Gal d 1 maintained at 4°C.

To understand how heat affects allergen structure and function, we first analyzed purified Gal d 1 and Gal d 2 proteins before assessing complex extracts. This allowed detailed characterization of molecular changes. SDS‐PAGE analysis showed temperature‐dependent structural modifications, with changes for Gal d 2 beginning at 75 °C (Figure 1A) and at 85 °C for Gal d 1 (Figure 1B). Higher temperatures (85°C–95 °C) intensified these effects, indicated by shifts to higher molecular weight bands, increasing with temperature and heating duration.

ELISA measurements confirmed a temperature‐dependent reduction in immunoreactivity. For Gal d 2, signal decline began at 65°C–75°C within 10 min, with complete loss at 95 °C (Figure 1C). Gal d 1 showed marked signal loss starting at 85 °C, with complete loss at 95 °C after 20 min (Figure 1D), suggesting significant conformational and epitope disruption. Similar reductions were observed in heat‐treated WEP samples, confirming substantial loss of detectable Gal d 2 and Gal d 1 proteins (Figure 1C,D). Extended heating at 95 °C for 1 h nearly eliminated detectable signal (data not shown).

To further examine structural changes, CD spectra of Gal d 2 were recorded (Figure 1E). Native Gal d 2 exhibited a positive peak near 190 nm and a negative peak between 208 and 222 nm, indicating α‐helical structures. Quantifying ellipticity at 222 nm across 40–90 C (using 30 C as reference) revealed major structural changes above 70 C, most pronounced at 90 C. Nano differential scanning fluorimetry showed an inflection temperature of 81.7 C (Figure 1F), indicating the transition to protein unfolding. Below 70 °C, denaturation was slower, consistent with CD findings.

### 3.2 Heat treatment reduces IgE binding activity of egg allergens

IgE ELISA demonstrated that increasing temperature (80°C–100 °C) reduced IgE binding to Gal d 2, with stronger effects at higher temperatures (Figure 1G). Longer heat exposure further decreased IgE binding, underscoring the combined impact of temperature and duration on allergenicity. Similarly, IgE binding to Gal d 1, measured using sera from three egg‐allergic patients, showed significant reduction after heating, compared to the unheated control stored at 4 °C (Figure 1H).

### 3.3 Study population

**FIGURE 2:**
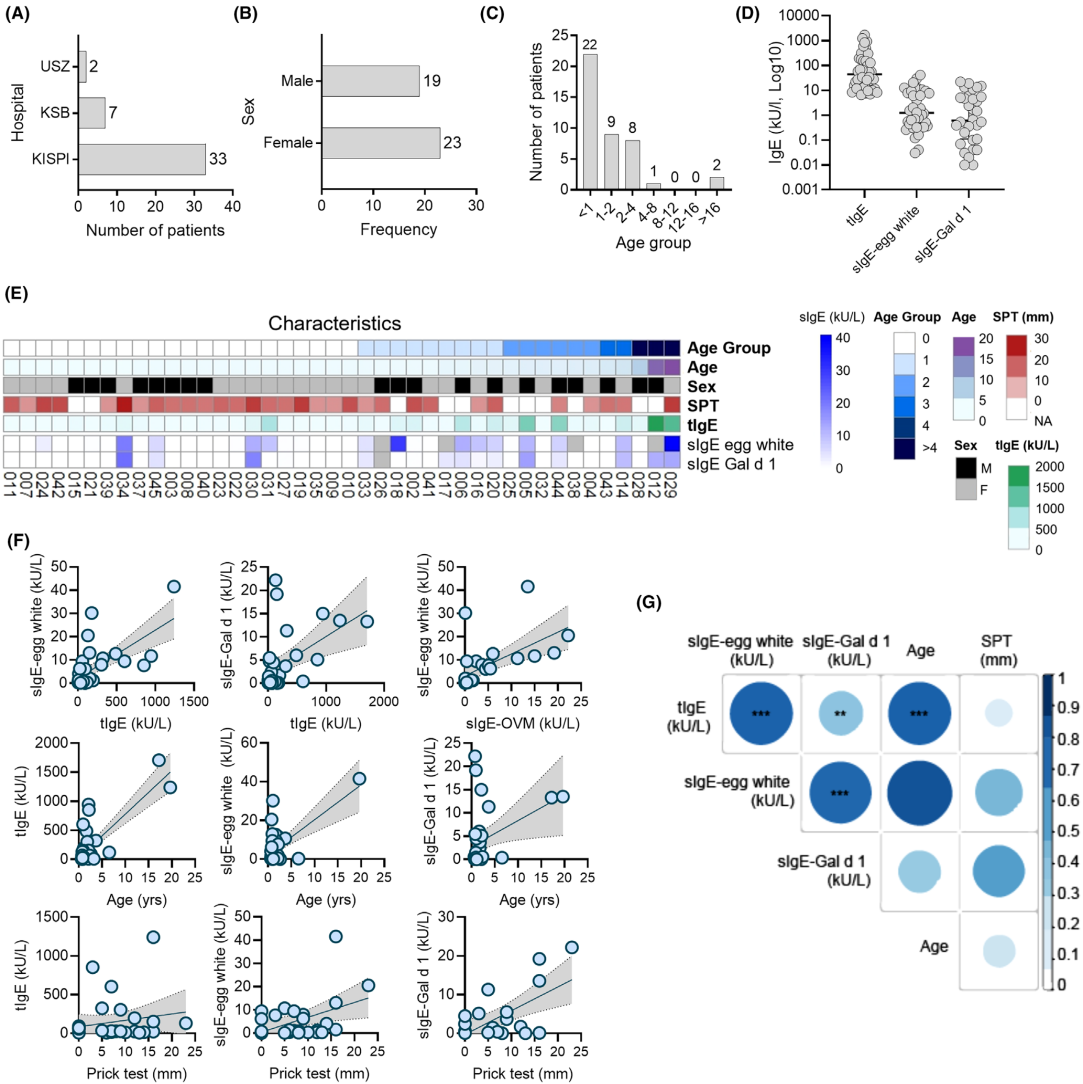
Patient characteristics and IgE antibody profiles (A) Distribution of hospitals providing patient blood samples. (B) Sex distribution of the study cohort. (C) Age distribution of patients, grouped by age categories (in years). (D) Serum IgE profiles in the study cohort: distributions of total IgE (tIgE, left), egg white‐specific IgE (sIgE‐egg white, middle), and Gal d 1‐specific IgE (sIgE‐Gal d 1, right), shown with medians and 95% confidence intervals (CI). (E) Heat map summarizing clinical and immunological characteristics of the individual patients. Each column represents a single patient; rows denote sex, age, skin prick test wheal diameter (mm), total serum IgE levels (kU/L), and allergen‐specific IgE levels (kU/L) to egg white and ovomucoid (Gal d 1). (F) Individual scatter plots illustrating the correlations between total IgE (tIgE), allergen‐specific IgE (egg white sIgE‐egg white and ovomucoid sIgE‐Gal d 1), patient age, and skin prick test wheal diameters. (G) Correlation matrix depicting pairwise associations between clinical and immunological variables. The size and color intensity of the circles reflect the strength and the direction of the correlations; larger circle = stronger correlation, darker color = more positive correlation. The color bar indicates the correlation coefficient r^2^. Statistically significant correlations are indicated: **p < .01, ***p < .001.

Forty‐two patients with diagnosed egg allergy and two non‐allergic controls were included to test basophil activation with native and heat‐denatured WEP. Diagnosis was confirmed by the treating allergist at each clinical site. Of the participants, 33 were from the Children's Hospital Zurich (KISPI), seven from *Kantonsspital Baden* (KSB), and two from University Hospital Zurich (USZ) (Figure 2A). Blood samples were collected between November 2023 and November 2024. Among the 42 patients, 23 (55%) were females and 19 (45%) males (Figure 2B). Twenty‐two (52%) were younger than 1 year, nine (21%) were aged 1–2 years, and eight (19%) were 2–4 years old (Figure 2C). One patient (2%) was between 4 and 8 years, and two patients (5%) were over 16 years. The median age was 11.5 months (range: 6.9 months to 19.6 years).

### 3.4 IgE levels and sensitization patterns

The clinical sites provided data on total IgE (tIgE) and allergen‐specific IgE (sIgE) to egg white proteins (f1, sIgE‐egg white, *n* = 38) and Gal d 1 (f233, sIgE‐Gal d 1, *n* = 41) (Figure 2D). The median tIgE level was 44.45 kU/L (range: 6.6–1707.0 kU/L). Among tested patients, 27 (71%) had egg white‐sIgE levels >0.35 kU/L, with a median of 1.24 kU/L (0.03–41.55 kU/L). Gal d 1‐sIgE levels >0.35 kU/L were found in 21 patients (51%), with a median of 0.60 kU/L (0–22.20 kU/L).

To visualize clinical and immunological heterogeneity across the cohort, a heat map was created (Figure 2E), summarizing individual data on sex, age, skin prick test wheal size, tIgE, and sIgE to egg white and ovomucoid. This highlighted variable sensitization patterns and demographic characteristics.

A strong positive correlation was found between age and tIgE (*p* = .0004, Figure 2F,G), though no correlation was seen between age and sIgE levels. Significant positive correlations were observed between tIgE and sIgE‐egg white (*p* = .0002), tIgE and sIgE‐Gal d 1 (*p* = .003), and sIgE‐egg white and sIgE‐Gal d 1 (*p* < .0001), as shown in scatter plots (Figure 2F) and the correlation matrix (Figure 2G).

### 3.5 Heat‐denaturation of egg allergens reduced degranulation of basophils

All blood samples were stimulated with native and heat‐denatured WEP and then analyzed for soluble leukotriene (sLT) release using the CAST ELISA. Typically, 3–4 WEP concentrations (e.g., 10, 1, 0.1, and 0.01 μg/mL) were tested to assess patient reactivity, depending on the blood volume available.

**FIGURE 3:**
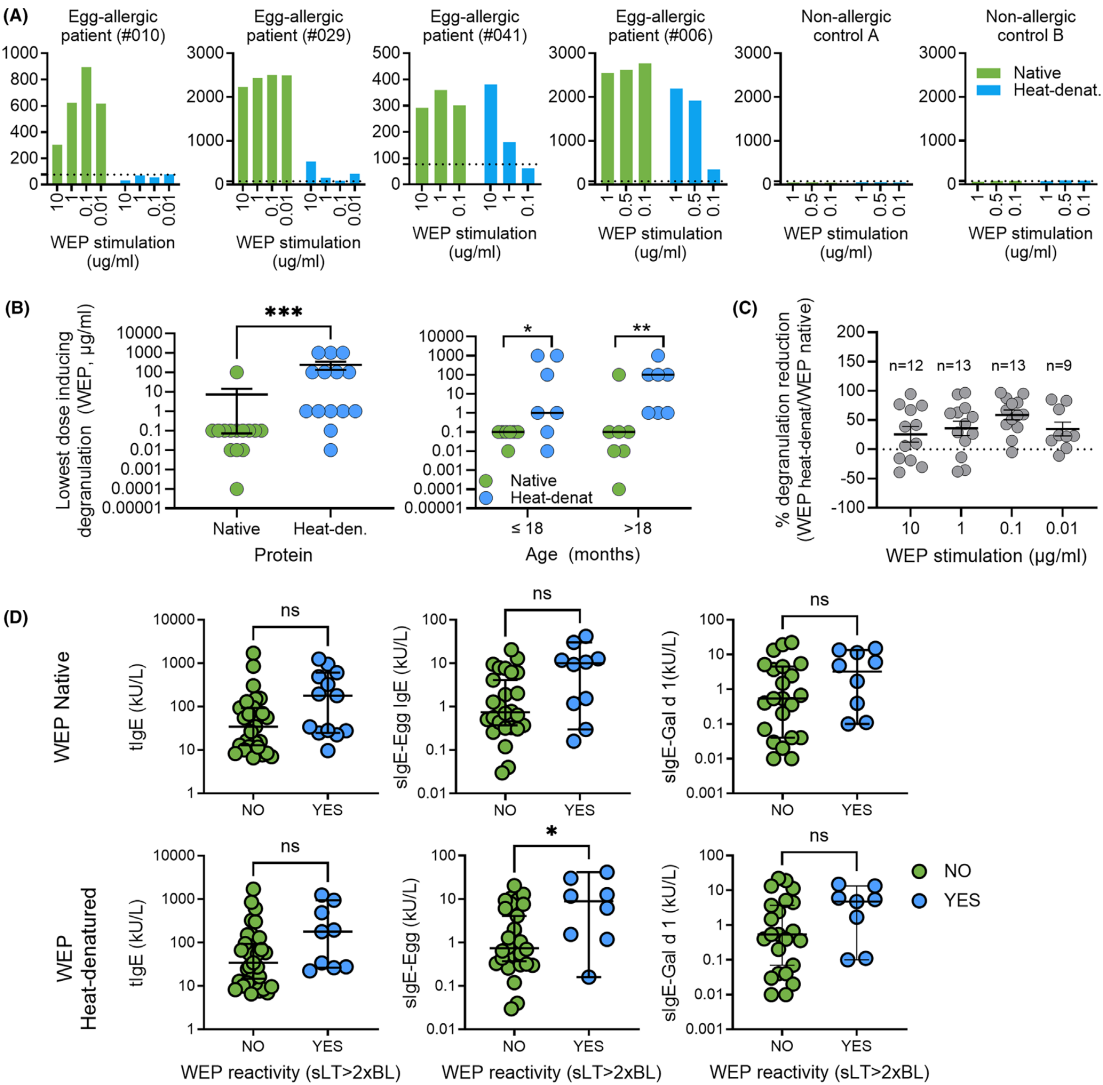
Leukotriene release and IgE characteristics in response to whole egg protein (WEP) stimulation (A) Representative examples of sLT release from patient blood cultures stimulated in vitro with either native WEP (green bars) or heat‐denatured WEP (95°C; blue bars). Supernatants were analyzed using CAST‐ELISA. Data shown from three representative egg‐allergic patients (patient #010, #025, #041, and #006) and two non‐allergic controls (A and B), tested side‐by‐side. (B) Minimal protein concentration required to elicit degranulation responses in blood cells stimulated with native or heat‐denatured WEP and as measured for all 14 basophil‐reactive patients (left panel) and stratified to patient younger or older than 18 months (right panel). Mann–Whitney *U* tests were applied. The data were analyzed for the pool of all 14. (C) Reduction in degranulation was expressed as the percentage decrease in response to heat‐denatured WEP relative to native WEP, using the formula: 100‐(heat‐denatured WEP/native WEP) × 100. (D) Relationship between total and specific IgE levels and blood‐cell reactivity to native (upper panel) and heat‐denatured (lower panel) WEP. Reactivity was defined as leukotriene release exceeding twice the spontaneous release of unstimulated cells (baseline; Mann–Whitney test; **p < .01).

The absolute degree of sLT release varied substantially among patients, and Figure 3A illustrates the sLT release in response to native WEP (green bars) and heat‐denatured (blue bars) WEP in four representative patients with egg allergy and in two non‐allergic controls. In blood from two egg‐allergic patients (#010 and #029), native WEP elicited a strong sLT release, while stimulation with heat‐denatured WEP hardly stimulated any sLT release. In another two egg‐allergic patients (#041 and #006), native WEP again stimulated strong sLT release, independent on the allergen concentration, whereas heat‐denatured WEP induced a weaker and a dose‐dependent sLT release. No sLT release was observed in blood from patients not allergic to egg.

The analysis of blood samples from all 42 patients revealed that 14 patients (33%) reacted to native WEP, while 28 patients (66%) did not reach the threshold reaction. In contrast, only 10 patients (23%) reacted to heat‐denatured WEP, while 32 patients (76%) remained non‐reactive. Out of the 14 patients who reacted to native WEP, five (43%) did not react to heat‐denatured WEP, while all patients that reacted to heat‐denatured WEP also reacted to native WEP. Among the 28 patients who did not reach the threshold reaction to native WEP, the majority were also non‐reactive to heat‐denatured WEP, with only two patients showing reactivity. Furthermore, a significantly higher concentration of heat‐denatured WEP was required to trigger degranulation and sLT release than after stimulation with native WEP, regardless of whether all study participants with positive responses to native WEP were pooled or stratified by age (<18 months, *n* = 7; ≥18 months, *n* = 7) (Figure 3B). This is generally reflected in the relative reduction in degranulation with heat‐denatured WEP (Figure 3C). The majority of patients showed consistently weaker reactivity to heat‐denatured WEP compared with native WEP, with responses increasing at higher doses of the stimulating WEP. These findings confirm that, at the cohort level, heat denaturation of WEP reduces allergenic potency, particularly at lower stimulation doses.

To investigate whether WEP‐reactive patients exhibited a specific IgE pattern, total IgE (tIgE) and allergen‐specific IgE levels (sIgE‐egg white and sIgE‐Gal d 1) were analyzed in relation to WEP reactivity (Figure 3D). The median levels of sIgE‐egg white were significantly higher in patients whose blood cells reacted to heat‐denatured WEP (8.9 kU/L) compared to those who were non‐reactive to heat‐denatured WEP (0.74 kU/L). Overall, a trend of higher tIgE, sIgE‐egg white, and sIgE‐Gal d 1 levels was observed in patients who exhibited reactivity to both native and heat‐denatured WEP.

## 4 DISCUSSION

This study aimed to explore how heat treatment affects the major egg allergens and evaluate their potential use as safer options for allergen immunotherapy in patients with egg allergy. Heat‐denaturation has long been considered a method to reduce allergen IgE binding and therefore make them less likely to trigger severe allergic responses.^33^, ^35^ Indeed, approximately 75% of children with milk^44^ or egg^38^ allergy tolerate heated or baked milk and egg products, suggesting that heating can modify protein structures and thereby alter the allergenicity of allergens. Our findings reinforce this concept, showing that heat‐induced structural changes in these allergens can significantly reduce their ability to bind to IgE and can have a functional consequence on basophil activation.

Heat treatment of Gal d 2, caused substantial structural changes, with increasing temperatures resulting in shifts in molecular weight and alterations in the protein's secondary structure. Even moderate heating (65°C–75°C) impacted the allergen's ability to bind IgE, but these changes were most pronounced at 85°C–95°C, where IgE‐binding activity was markedly reduced. Although Gal d 1 is generally considered heat‐stable,^45^ we observed that prolonged heating above 85°C induced detectable structural modifications and a measurable decrease in IgE reactivity. The formation of high molecular weight aggregates in Gal d 1 and Gal d 2 following extended heating also aligns with the behavior observed for other proteins.^33^, ^46^, ^47^ Moreover, heat‐denaturation of egg proteins caused a loss of detectable levels of Gal d 1 and Gal d 2, confirming that the process significantly reduces allergenic load, even in a protein mixture. Importantly, the CD spectra suggested that while some degree of structural rearrangement occurs, egg allergens are not fully denatured. Moreover, complete denaturation may not be required, as a partial unfolding appears sufficient to reduce IgE binding and may preserve immunogenic properties essential for inducing potentially neutralizing IgG antibodies. This balance is critical for improving the safety of OIT, where reduced allergenicity must be paired with the ability to elicit an effective immune response.

Of note, IgE antibodies can target both conformational and linear epitopes, with persistent allergies being more commonly associated with IgE recognition of sequential epitopes.^28^ Linear epitopes, especially in heat‐stable allergens like ovomucoid (Gal d 1), often remain intact after heating, and binding to these sequential epitopes has been associated with persistent egg allergy and reduced tolerance to baked products.^25^, ^45^ More broadly, heat treatment may primarily disrupt conformational IgE epitopes, while linear T‐cell epitopes may remain intact.^25^, ^48^ Evaluating T‐cell reactivity will therefore be important in determining the immunotherapeutic potential of heat‐denatured allergens.

While previous studies have focused on the structural stability and IgE‐binding capacity of egg allergens following heat treatment, our study extends these observations by examining functional immune responses, particularly basophil activation. This functional assessment is critical because it reflects the actual cellular activation potential of allergens in sensitized individuals. In our cohort of egg‐allergic patients, 42.8% of the patients whose leucocytes reacted to native WEP showed no response to heat‐denatured WEP, suggesting that heating substantially impairs the ability of egg allergens to trigger basophil degranulation. Interestingly, among the patients who did respond to heat‐denatured WEP, significantly higher allergen doses were required to induce degranulation, further underscoring the reduced allergen potency of heat‐denatured allergens. Notably, we observed that patients with higher levels of egg‐white‐specific IgE were more likely to react to heat‐denatured allergens. Correlations between total IgE (tIgE), specific IgE to egg (sIgE‐egg white), and ovalbumin (sIgE‐Gal d 1) indicated that, among patients with reactivity, higher sensitization profiles were associated with stronger basophil responses to both native and heat‐denatured allergens. When stratifying responders by age (<18 months vs. ≥18 months), the reduction in degranulation with heat‐denatured WEP remained evident in both groups, indicating that the attenuated allergenic potency of heat‐denatured proteins is consistent across age strata. However, as older children are less likely to achieve spontaneous tolerance and often present with more persistent disease, this subgroup may represent a clinically more relevant population for future therapeutic strategies. Together, these findings highlight the significance of individual sensitization profiles and the crucial role of protein structure in allergenic activity. Our results demonstrate that while heat treatment can attenuate allergenicity, its impact varies across patients, likely depending on their sensitization to linear versus conformational epitopes. The variability in patient responses to denatured allergens highlights the need for more personalized assessments when considering heat‐denatured allergens for immunotherapy. Functional assays like basophil activation tests could help identify patients who are more likely to tolerate these modified proteins, thereby improving the safety and efficacy of OIT. Additionally, these assays may help identify which patients are appropriate candidates for an oral food challenge to assess current tolerance to baked egg, and ultimately guide the initiation of a food ladder approach for gradual dietary reintroduction.^49^

Similar to findings in other allergen sources, including bee venom, cat hair, grass pollen, birch pollen, and house dust mite, heat‐denatured egg allergens showed reduced IgE binding capacity. Importantly, previous studies have also shown that such denatured proteins can induce more favorable immune responses, including enhanced Th1‐type skewing and increased production of neutralizing IgG antibodies in animal models.^35^, ^46^ In mice, heat‐denatured allergens, despite structural modifications, retained recognition by human IgG4 at levels comparable to native allergens.^35^ These findings suggest that heat‐denatured egg allergens may retain immunotherapeutic value with a lower risk of allergic reactions, yet capable of engaging immune pathways that contribute to long‐term desensitization. Although many egg‐allergic children who tolerate baked egg eventually progress to tolerating less‐heated or raw forms, this progression is not uniform, with some patients remaining reactive to less‐denatured egg proteins.^39^ Our findings may also inform the design of future OIT protocols. While current egg OIT approaches often begin with baked or extensively heated egg products, these are not standardized and can vary in allergen content. By contrast, heat‐denatured egg protein preparations could be manufactured under controlled conditions to provide reproducible allergen doses with reduced allergenic potency. Such preparations could be formulated into predefined dosing units, for example, sachets or capsules of protein powder, enabling dose escalation protocols comparable to those established for FDA‐approved peanut OIT,^50^, ^51^ which escalates doses under controlled conditions. In this way, heat‐denatured egg proteins may serve as safer and more standardized starting materials for OIT, forming either the initial steps of an “egg ladder” that gradually progresses toward less‐denatured and native egg proteins or as the agent used as an alternative to “baked‐egg OIT.”

In conclusion, our findings offer new insights into how thermal processing alters the structure and immunological activity of egg allergens. Heat‐induced modifications to Gal d 1 and Gal d 2 reduced IgE binding and decreased basophil degranulation, suggesting a lower allergenic potential. However, the degree of patient sensitization, particularly to sequential epitopes, remains a crucial determinant of residual reactivity. This study highlights the complexity of allergic responses and points to heat‐denatured allergens as promising, yet not universally safe, candidates for allergen immunotherapy strategies. Personalized assessment of IgE profiles and functional immune responses could enhance the safety and effectiveness of OIT protocols. A limitation of this study is that the cohort is dominated by very young children who tend to have mild allergy and a high rate of outgrowing.^52^ Since egg sensitization in childhood is a dynamic process, some patients may have been in the process of developing partial tolerance while being treated. Many were managed according to a stepwise “food ladder” approach with gradual introduction of baked or cooked egg. However, the cohort reflects both the epidemiology of egg allergy, which is predominantly a pediatric condition, and the fact that very few adult patients with egg allergy present at our clinics. Adults with persistent egg allergy often adapt their nutritional habits to avoid egg‐containing foods, which reduces their need for specialist evaluation. While this limits the generalizability of our findings to adults, the cohort remains representative of most egg‐allergic patients encountered in clinical practice. Future studies should also investigate the stability of denatured allergens during gastrointestinal digestion to fully evaluate their potential as immunotherapeutic agents in vivo. To this end, prior work demonstrates that heat‐denatured ovalbumin and ovomucoid show enhanced digestibility and reduced transport across intestinal epithelium, and do not provoke basophil activation or anaphylaxis in sensitized models.^25^ Heat‐coagulated ovalbumin is more susceptible to peptic digestion than its native form,^53^ and egg white gels heated to 95°C display significantly improved proteolytic degradation in adult in vitro digestion systems.^54^ These data support the clinical safety rationale for using heat‐denatured egg proteins in OIT strategies. Together, these findings support a stepwise, individualized approach to desensitization that incorporates heat‐denatured egg proteins as safer starting materials for OIT.

## AUTHOR CONTRIBUTIONS

**Marta Paolucci:** Writing – original draft; writing – review and editing; formal analysis; data curation; methodology; investigation; conceptualization. **Maria Breiding:** Methodology. **Macsmeila Dietrich:** Investigation. **Jana G. Meyer:** Investigation; data curation. **Agathe Duda:** Investigation. **Alice Köhli:** Methodology; writing – review and editing. **Claudia C. V. Lang:** Methodology; writing ‐ review and editing. **Sayeh Agah:** Data curation; writing – review and editing; methodology; investigation; formal analysis. **Sabina Wuenschmann:** Investigation; writing – review and editing; data curation; formal analysis. **Klaus Eyer:** Methodology; writing ‐ review and editing; formal analysis. **Thomas M. Kündig:** Project administration; funding acquisition. **Johannes Trück:** Writing – review and editing; methodology. **Pål Johansen:** Conceptualization; funding acquisition; writing – original draft; methodology; supervision; project administration; writing – review and editing; data curation.

## CONFLICT OF INTEREST STATEMENT

Pål Johansen and InBio received financial support from Hanimune Therapeutics Inc. that is developing and selling foods for prevention or treatment of food allergy. Sayeh Agah and Sabina Wuenschmann are employed at InBio that provided materials used in the study. The other authors disclose no conflict of interest.
